# Detection and recognition thresholds for five basic tastes in patients with mild cognitive impairment and Alzheimer’s disease dementia

**DOI:** 10.1186/s12883-020-01691-7

**Published:** 2020-03-26

**Authors:** Minoru Kouzuki, Junya Ichikawa, Daiki Shirasagi, Fumiya Katsube, Yuuki Kobashi, Hideki Matsumoto, Huichia Chao, Shintaro Yoshida, Katsuya Urakami

**Affiliations:** 1 Department of Biological Regulation, School of Health Science, Faculty of Medicine, Tottori University, 86 Nishi-cho, Yonago, 683-8503 Japan; 2 Research Institute for Bioscience & Fine Chemicals, Ajinomoto Co., Inc., 1-1, Suzuki-Cho, Kawasaki-Ku, Kawasaki-Shi, 210-8681 Japan

**Keywords:** Alzheimer disease, Mild cognitive impairment, Gustatory function, Gustatory test

## Abstract

**Background:**

Patients with Alzheimer’s disease dementia (ADD) are thought to exhibit taste disorders; however, this has not been extensively studied. We investigated gustatory functions and factors affecting taste in patients with ADD or mild cognitive impairment (MCI) and in non-demented controls (NDCs) and evaluated associations between cognitive impairment and gustatory functions.

**Methods:**

We recruited 29 patients with ADD, 43 with MCI, and 14 with NDCs. We obtained medical and medication history, measured salivary secretion volumes, and performed cognitive function tests, blood tests, whole-mouth gustatory tests, and dietary and gustatory questionnaires.

**Results:**

Patients with ADD showed significantly higher recognition threshold values than NDCs (*p* < 0.05). Many individuals did not recognize umami at the maximum concentration, and this happened more frequently in patients with ADD or MCI than in NDCs. Evaluation items other than cognitive function tests did not show significant differences among the groups, but many individuals had decreased salivation, low serum zinc levels, and were on multiple medications. We found a significant correlation between recognition threshold and age (r = 0.229, *p* < 0.05) and cognitive function test score (*r* = 0.268, *p* < 0.05).

**Conclusions:**

Patients with ADD showed impairment of gustatory function. Gustatory impairment in patients with MCI could not be confirmed. However, many individuals with MCI did not recognize umami, either. Our results suggest that taste disorders in elderly people with cognitive decline occur independently of factors affecting taste such as salivation, zinc levels, or prescription drugs.

**Trial registration:**

The study was registered in the UMIN Clinical Trials Registry on February 10, 2017, with reference number UMIN000026087.

## Background

Olfactory function declines in patients with Alzheimer’s disease dementia (ADD) [1–4], and hearing loss is a risk factor for dementia onset [5–7], indicating that there are associations between sensory disorders and dementia. In terms of the sense of taste, gustatory function has been shown to be decreased in patients with ADD [8–10], and 81.4% of patients with ADD show some eating disturbance, such as changes in appetite and eating habits [11]. Gustatory tests include the filter-paper disc method [12], the taste-strip test [13, 14], electrogustometry [12, 15], and the whole-mouth gustatory test [16, 17]. The filter-paper disc method, the taste-strip test, and the whole-mouth gustatory test are used to evaluate the identity of taste (taste quality), whereas electrogustometry can evaluate taste thresholds. A study based on the filter-paper disc method and electrogustometry has suggested that patients with ADD may exhibit taste disorder concomitantly with brain atrophy and neurodegeneration [8]. Also, another study using taste strips reported reduced taste function in patients with mild cognitive impairment (MCI), which is a pre-dementia state [9]. However, other reports using filter-paper discs have found no deterioration of the taste function in patients with ADD [18], and there was no detection threshold disorders for sweet and sour tastes in whole-mouth gustatory tests [19]. Some diverging results may be explained by the use of different evaluation methods. For example, when patients with ADD were evaluated by filter-paper discs and then by whole-mouth gustatory tests, the whole-mouth gustatory test indicated taste deterioration, but the filter-paper discs did not [20]. In addition, comparing studies is difficult due to different taste solution concentrations and results varying between evaluations regarding detection or recognition thresholds. Moreover, many studies have evaluated only four basic tastes (sweet, salty, sour, and bitter) and have not evaluated the fifth basic taste, umami. Inability to sense umami is thought to lead to loss of appetite [21], and umami evaluation is important to assess appetite in people with dementia. In addition, other factors affecting taste function like prescription drugs, saliva secretion, and serum zinc levels need to be considered in the interpretation of taste function tests [22–26]. Also, few studies have examined taste in patients with MCI, and, thus, it is unclear whether taste function is impaired in these patients.

The aim of this study was to conduct a detailed investigation into the detection and recognition of the five basic tastes in the taste function of patients with ADD and MCI, which has not been sufficiently investigated in previous studies. In this research, we adopted whole-mouth gustatory tests that evaluate the taste function in the whole oral cavity, and tested patients with ADD or MCI, and non-demented controls (NDCs) using diluted solutions of the five basic tastes. In addition, we investigated several factors related to gustatory function to consider the association of gustatory function with cognitive decline.

## Methods

### Patients

Between May 2017 and December 2018, we recruited patients with ADD (*n* = 29) or MCI (*n* = 43), and NDCs (*n* = 14), from the outpatient clinic of Shinsei Hospital (Kurayoshi, Japan). All patients underwent general physical and neurological examinations, neuropsychological assessments, such as the Geriatric Depression Scale 15-item version, the Mini-Mental State Examination (MMSE), and/or the Alzheimer’s Disease Assessment Scale-Cognitive Subscale (ADAS-Cog), laboratory tests, and brain imaging studies, such as brain computed tomography (CT), magnetic resonance imaging, and single photon emission CT, to exclude other potential causes of dementia. We included patients with ADD that met the diagnostic criteria of the Diagnostic and Statistical Manual of Mental Disorders, fifth edition [27] and patients with MCI that met the following diagnostic criteria [28]: (i) memory complaint, preferably corroborated by an informant, (ii) objective memory impairment for age, (iii) relatively preserved general cognition for age, (iv) essentially intact activities of daily living, and (v) not demented, or NDCs without evidence of cognitive impairment or psychiatric disorders at the time of clinical and neuropsychological assessments. We excluded patients who had already been diagnosed as having taste disorders.

The ethics committee of Tottori University (Yonago, Japan) and Ajinomoto (Tokyo, Japan) approved the design of this study (approval number: 1701B073), which was conducted by following the tenets of the Declaration of Helsinki. We registered the study in the UMIN Clinical Trials Registry (UMIN000026087). We explained the research protocol to the patients and guardians, who provided written informed consent before participation.

### Procedures

We performed gustatory and cognitive function tests on the subjects, measured salivary flow, height, weight, blood pressure, performed blood tests, and asked participants to answer dietary and gustatory questionnaires within 1 month after obtaining the participants’ consents. If blood samples were collected during routine medical examination 1 month before or after the gustatory test, blood tests were performed using that sample. We determined the number of prescribed medications taken by the participants and their medical history (hypertension, dyslipidemia, and diabetes mellitus) from medical record data.

### Gustatory test

We used the whole-mouth method by 13 stepwise two-fold dilution series of five basic tastes prepared according to previous studies: sweet (40–0.0098% sucrose), salty (20–0.00488% sodium chloride), umami (2.0–0.00049% monosodium L-glutamate [MSG]), sour (1.6–0.00039% tartaric acid), and bitter (0.1–0.00002% quinine hydrochloride) [16, 29]. Gustatory tests were performed according to the procedure in a previous study [16]. The patients first rinsed their mouths with water, and then we applied 1 mL of a taste solution at the lowest concentration onto their tongue, and asked the patients to spread it around in their entire mouth before spitting it out. For each solution tested, the participants chose one of the following seven possible answers: sweet, salty, umami, sour, bitter, unidentifiable taste, or no taste. If the patient could not correctly identify the taste, in the next trial, we used the solution in the same taste category with the next higher concentration. If the patient answered correctly, we tested a different taste solution. Before testing another solution, the patients rinsed their mouths thoroughly until the previous taste disappeared. We applied the taste solutions in the following order; sweet, salty, umami, sour, and bitter. We did this to ensure that the bitter taste, which tends to remain in the oral cavity for a long time, was applied last. With increase in the concentration of the taste solution, the detection and recognition thresholds were recorded. We defined the detection threshold as the concentration at which the taste could be detected, and the recognition threshold as the concentration at which the taste could be recognized. To determine the detection threshold, we adopted the lowest concentration at which the participant answered other than “no taste.” Even if participants answered that they tasted something, if they answered “no taste” when the concentration was increased after that, the previous answers were cancelled. Patients who detected the lowest concentration received 1 point. Patients who detected the highest concentration received 13 points. Patients failing to detect the highest concentration received 14 points. For the recognition threshold, we adopted the lower concentration at which the participant answered correctly twice in a row. The scoring method for the recognition threshold was set from 1 to 14 as well as with the detection threshold.

### Cognitive function test

To assess cognitive function, we used the Touch Panel-type Dementia Assessment Scale (TDAS) (Nihon Kohden Corporation, Tokyo, Japan) [30]. TDAS is a modified version of the ADAS-Cog [31] in which subjects enter their answers directly into a touch-panel computer following instructions. The nine examination items included “word recognition,” “following simple commands,” “visual–spatial perception,” “accuracy of the order of a process,” “naming fingers,” “orientation,” “money calculation,” “object recognition,” and “clock time recognition.” The validity of the TDAS has previously been tested and the results showed a significant association between TDAS and ADAS-cog [30]. Scores ranged from 0 (all correct answers) to 101 points (all incorrect answers). A previous study showed that the average TDAS score was approximately 20 points for patients with mild to moderate ADD patients with an average MMSE score of 20 points, approximately 7 points for patients with MCI, and approximately 3 points for patients without cognitive impairment [2]. In contrast, the average TDAS score was shown to be approximately 50 points in individuals with moderate to severe dementia residing in a hospital or nursing home [32].

### Dietary and gustatory questionnaire

We designed a dietary and taste questionnaire. We focused on four items (enjoyment of the meal, deliciousness of the meal, strength of the flavor, and subjective gustatory function) to inquire about palatability of the foods and gustatory function. Each item was self-rated by the participants on a five-point scale (from 0 to 4 points, with 0 indicating the worst and 4 indicating the best).

### Measurement of salivary flow

We measured the saliva secretion volume at rest according to a published protocol [33]. We asked each participant to sit in a quiet room and not to swallow but to continually spit into a receiving tube over a period of 5 min. We then collected the (resting) whole saliva sample. We estimated the volume of saliva by weighing the tubes before and after collection. Different studies have provided different results in terms of the cutoff value for salivary gland hypofunction [33–35]. We used a cutoff value of < 0.2 mL/min considering the boundary area.

### Blood tests

Collected blood samples were centrifuged to obtain serum, and the serum samples were immediately stored in polypropylene containers at − 80 °C until analysis. The levels of total protein (reference: 6.7–8.3 g/dL), albumin (reference: 3.8–5.2 g/dL), and zinc (reference: 64–111 μg/dL) in the serum were measured by LSI Medience Corporation (Tokyo, Japan).

### Statistical analysis

We performed all statistical analyses using the SPSS statistical software (version 25; IBM Japan, Tokyo, Japan). We performed Shapiro–Wilk tests to assess data distributions. We assessed differences in the baseline demographics and background characteristics of the participants using one-way analysis of variance (ANOVA), the Kruskal–Wallis test, or the chi-squared test as appropriate. For multiple comparisons, we used the Tukey method or Bonferroni correction. We compared gustatory test scores among the three groups by one-way ANOVA or the Kruskal–Wallis test. The Tukey method or Bonferroni correction was used for multiple comparisons of the degree of change in each group. Furthermore, if the data were normally distributed, the gustatory test scores were compared among the three groups by an analysis of covariance (ANCOVA), with significantly different background characteristics among the three groups as covariates. Consequently, only age was used as a covariate. For multiple comparisons, we used the Bonferroni correction. We applied Pearson’s correlation coefficient or Spearman’s correlation coefficient to assess correlations between the gustatory test scores and the results of the other tests. All statistical significance tests were two-sided, and we considered alpha-levels of 0.05 as indicative of statistical significance.

## Results

### Clinical characteristics

Table 1 summarizes the characteristics of the participants. Patients in the ADD group were significantly older than those in the MCI and NDC groups (both *p* < 0.05). The TDAS scores were significantly higher in patients with ADD than in those with MCI or NDCs (both *p* < 0.05). The scores were also significantly higher in MCI compared with those in NDCs (*p* < 0.05). Patients with ADD demonstrated a median TDAS score (interquartile range) of 18 (11–23) points, which suggests mild to moderate ADD. For the other items, we found no significant differences between the three groups.

**Table 1:** Characteristics of patients

	ADD	MCI	NDC	*P* value
Demographics				
Number, n	29	43	14	-
Gender (M:F), n	9:20	15:28	3:11	0.641
Age (years), median (IQR)	83.0 (80–87)	79.0 (75–84)	74.0 (72–78)	0.002 ^a^
Hypertension, %	51.7	41.9	35.7	0.557
Dyslipidemia, %	24.1	30.2	28.6	0.851
Diabetes mellitus, %	10.3	7.0	14.3	0.696
Number of medications, median (IQR)	5.0 (1–6)	4.0 (0–7)	5.0 (3–7)	0.490
Smoking, %	0	0	7.1	0.074
Drinking, %	24.1	11.6	14.3	0.361
Physical examinations, median (IQR)				
Height (cm)	150.1 (146–160)	151.0 (147–161)	151.5 (148–157)	0.809
Weight (kg)	50.6 (45–60)	49.5 (45–58)	55.2 (50–58)	0.195
Body mass index (kg/m^2^)	22.4 (20–25)	21.5 (20–23)	22.1 (21–25)	0.150
Systolic blood pressure (mmHg)	134.0 (126–140)	130.0 (128–140)	134.0 (126–141)	0.913
Diastolic blood pressure (mmHg)	74.0 (72–80)	74.0 (71–80)	77.5 (73–86)	0.195
Salivary secretion volume (mL/5 min)	0.8 (0.4–1.4)	0.7 (0.4–1.1)	0.9 (0.2–1.4)	0.819
Blood tests, median (IQR)				
Total protein (g/dL), ref: 6.7–8.3	7.0 (6.6–7.4)	6.9 (6.6–7.2)	6.8 (6.6–7.3)	0.698
Albumin (g/dL), ref: 3.8–5.2	4.0 (3.8–4.2)	4.0 (3.8–4.2)	4.1 (4.0–4.3)	0.426
Zinc (μg/dL), ref: 64–111	65.0 (57–72)	62.0 (55–72)	63.0 (59–75)	0.620
Cognitive test, median (IQR)				
TDAS score	18.0 (11–23)	7.0 (5–12)	1.0 (1–4)	<0.001 ^b^
Questionnaires, median (IQR)				
Subjective gustatory function	4.0 (4–4)	4.0 (4–4)	4.0 (4–4)	0.891
Enjoyment of the meal	4.0 (4–4)	4.0 (3–4)	4.0 (4–4)	0.080
Deliciousness of the meal	4.0 (4–4)	4.0 (4–4)	4.0 (4–4)	0.254
Strength of flavor	4.0 (4–4)	4.0 (4–4)	4.0 (4–4)	0.443

Gender, hypertension, dyslipidemia, diabetes mellitus, smoking, and drinking ration were analyzed using the chi-squared test. Age, height, diastolic blood pressure, total protein, and zinc were compared by a one-way analysis of variance followed by the Tukey test. Items other than the above were compared by the Kruskal–Wallis test followed by the Bonferroni correction

*ADD* Alzheimer’s disease dementia, *MCI* mild cognitive impairment, *NDC* non-demented control, *IQR* interquartile range, *TDAS* Touch Panel-type Dementia Assessment Scale, *ref* reference values

^a^Significant difference between ADD and both MCI (*p* < 0.05) and NDC (*p* < 0.05)

^b^Significant difference between NDC and MCI (*p* < 0.05), between NDC and AD (*p* < 0.05), and between ADD and MCI (*p* < 0.05)

We found that in each group, participants took a median of 4 to 5 prescription drugs, and participants tended to have polypharmacy. As for body mass index (BMI), the median values were about 22 kg/m^2^ in each group, and we found no tendencies for thinness or obesity. We found that the salivary secretion volumes tended to be low in all groups (< 0.2 mL/min). The total protein, albumin, and zinc in the serum showed a tendency to be low in all groups compared with the reference values. The questionnaire survey showed that most of the participants did not complain about declines in gustatory function and were satisfied with their diet.

### Gustatory test

**Fig. 1:**
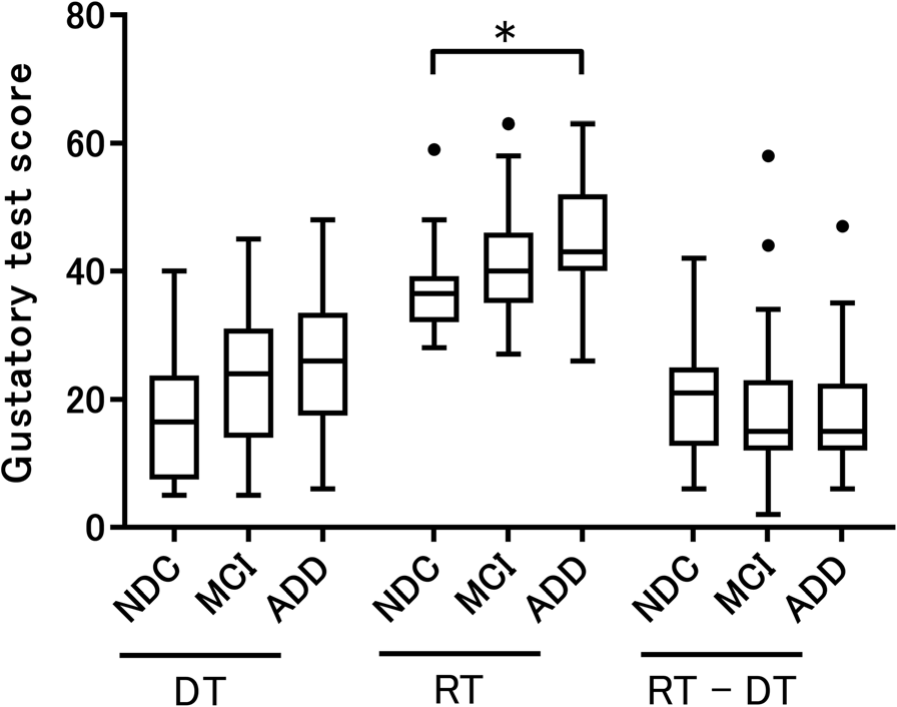
Results of gustatory tests in patients with Alzheimer’s disease dementia (ADD), mild cognitive impairment (MCI), and non-demented controls (NDCs). The recognition threshold (RT) of patients with ADD exhibited a significant increase compared with those in NDCs. However, detection threshold (DT) and RT minus DT (RT − DT) was not significant among three groups. The DT was compared by an analysis of covariance with age as a covariate followed by the Bonferroni correction. The RT and RT − DT were compared by the Kruskal–Wallis test followed by the Bonferroni correction. All data are indicated by box-and-whisker plots following the Tukey method (*p < 0.05)

**Fig. 2:**
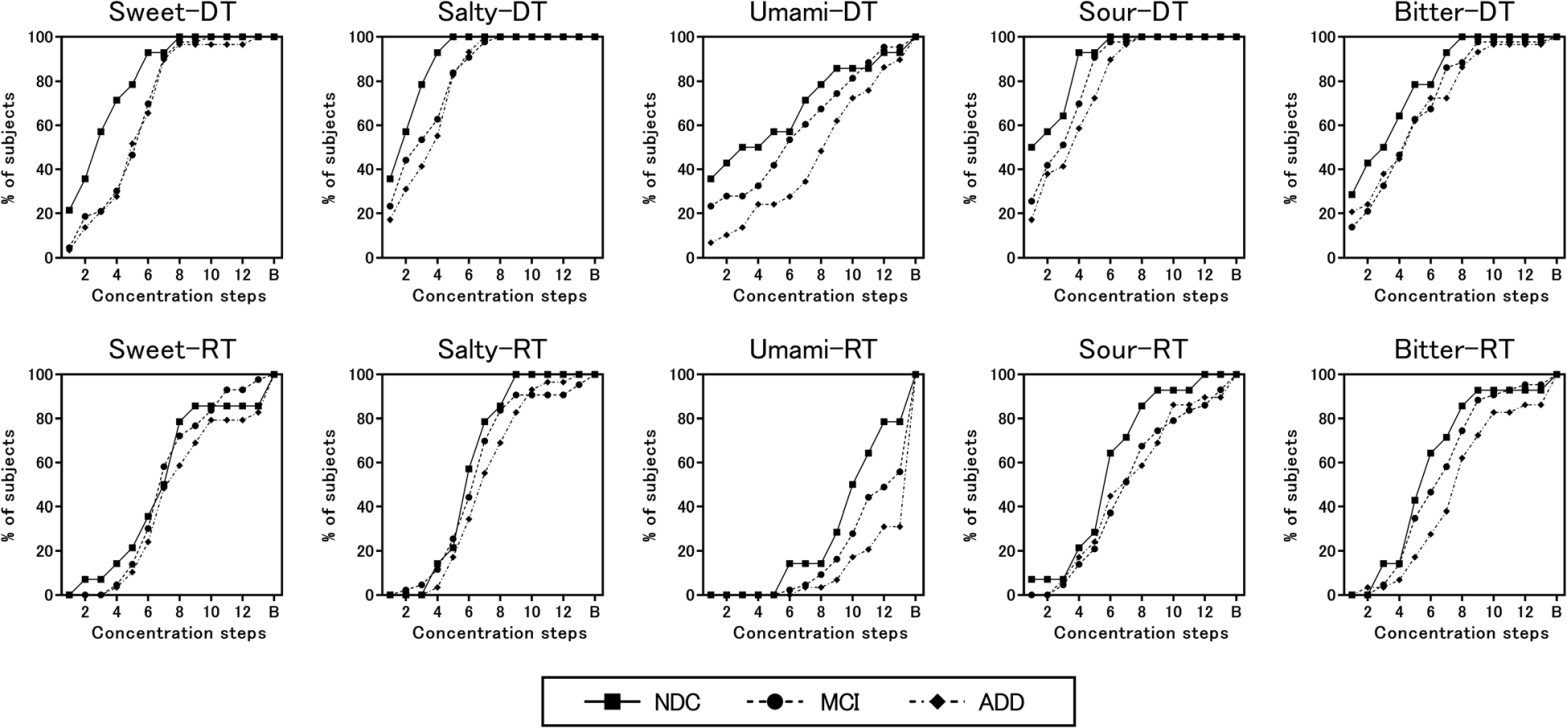
Cumulative detection threshold (DT) and recognition threshold (RT) curves in patients with Alzheimer’s disease dementia (ADD) or mild cognitive impairment (MCI), and that in non-demented controls (NDCs). If the participants could not detect and recognize a taste, even at the highest concentration, those results are indicated as “burst (B)”

Figure 1 shows results of gustatory tests (detection and recognition thresholds, and difference between the two). Patients with ADD showed significantly higher detection and recognition thresholds than NDCs according to an ANOVA and the Kruskal–Wallis test (both *p* < 0.05). Furthermore, we performed an analysis with the age of the subjects, which significantly differed among the three groups, as a covariate. In fact, all data should be performed with an age-adjusted analysis, but due to the complexity of dealing with non-normally distributed variables, we conducted an ANCOVA only for detection threshold, which was the normally distributed variable. The age-adjusted ANCOVA showed a tendency toward significance between patients with ADD and NDCs (*p* = 0.088). On the other hand, the difference between the recognition threshold and the detection threshold was not significantly different among the three groups. Table 2 shows the results for each taste. At the detection threshold of sweet, we found a significant increase in the ADD and MCI groups compared with the NDC group (both *p* < 0.05). In addition, at the recognition threshold of umami, we found a significant increase in the ADD group compared with the NDC group (*p* < 0.05). Figure 2 shows the cumulative curves of detection and recognition thresholds for each taste in each group. The detection and the recognition threshold curves of each taste tended to shift to the right in patients with MCI and ADD (curves at a higher concentration) compared with those in NDCs. However, the curves of umami were different from those of the other tastes (compared to other tastes, a higher concentration of umami was needed, in all groups). Table 3 shows data on the rate of failure to detect or recognize the highest concentration of a taste solution. In all groups, the percentage of “unrecognizable” responses for umami was the highest compared with other tastes.

**Table 2:** Results of gustatory test for each taste solution

	ADD		MCI		NDC		*P* value
	Median	IQR	Median	IQR	Median	IQR	
Sweet							
DT	5	4–7	6	4–7	3	2–4.8	0.026 ^a^
RT	8	7–10	7	6–9	7.5	6–8	0.481
RT - DT	3	0–4	1	1–4	3	2–6	0.123
Salty							
DT	4	2–5	3	2–5	2	1–3	0.075
RT	7	6–9	7	5.5–8	6	6–7	0.234
RT - DT	4	1–6	3	2–5	4	3–5	0.620
Umami							
DT	9	6–11	6	2–9.5	4	1–7.8	0.038 ^b^
RT	14	12–14	13	10–14	10.5	9.3–12	0.008 ^c^
RT - DT	4	2–7	6	2–9	6	3.5–7.8	0.623
Sour							
DT	4	2–6	3	1.5–5	1.5	1–4	0.064
RT	7	6–10	7	6–9.5	6	5.3–7.8	0.273
RT - DT	4	2–5	5	2–6	3.5	2.3–5.8	0.495
Bitter							
DT	5	3–8	5	3–7	3.5	1.3–5	0.325
RT	8	6–10	7	5–8.5	6	5–7.8	0.055
RT - DT	4	0–5	1	1–3	2	1–4	0.258

All data were compared via the Kruskal–Wallis test followed by the Bonferroni correction

*ADD* Alzheimer’s disease dementia, *MCI* mild cognitive impairment, *NDC* non-demented control, *IQR* interquartile range, *DT* detection threshold, *RT* recognition threshold

^a^ Significant difference between NDC and both MCI (*p* < 0.05) and ADD (*p* < 0.05)

^b^ No significant difference by multiple comparisons

^c^ Significant difference between NDC and ADD (*p* < 0.05)

**Table 3:** The rate of failure (i.e., burst) to detect or recognize the highest concentration of taste solution

	ADD		MCI		NDC	
	*n*	%	*n*	%	*n*	%
Burst of DT						
Sweet	0	0	0	0	0	0
Salty	0	0	0	0	0	0
Umami	3	10.3	2	4.7	1	7.1
Sour	0	0	0	0	0	0
Bitter	1	3.4	1	2.3	0	0
Burst of RT						
Sweet	5	17.2	1	2.3	2	14.3
Salty	0	0	2	4.7	0	0
Umami	20	69.0	19	44.2	3	21.4
Sour	3	10.3	3	7.0	0	0
Bitter	4	13.8	2	4.7	1	7.1

*ADD* Alzheimer’s disease dementia, *MCI* mild cognitive impairment, *NDC* non-demented control, *DT* detection threshold, *RT* recognition threshold

### Correlations between gustatory test results and other outcomes

Table 4 shows the results of the correlation analyses between the gustatory test results and age, the number of medications, BMI, the salivary secretion volume, blood test results, and the TDAS score. We found a significant correlation between the detection threshold and BMI (r = − 0.248, *p* < 0.05) and between the recognition threshold and age (*r* = 0.229, *p* < 0.05) and TDAS (*r* = 0.268, *p* < 0.05) in all patients. We also found a significant correlation between age and TDAS (*r* = 0.324, *p* = 0.002). In the analysis results for each diagnostic group, there was only a correlation between the detection threshold and zinc in the ADD group (*r* = − 0.390, *p* < 0.05).

**Table 4:** Correlation between gustatory test results and other test results

	Age	Medications	BMI	Salivation	Total protein	Albumin	Zinc	TDAS
All patients								
DT	0.186	−0.082	−0.248*	0.096	0.076	−0.168	−0.118	0.179
RT	0.229*	0.078	−0.067	0.177	0.132	−0.112	−0.065	0.268*
ADD								
DT	0.061	− 0.217	− 0.041	0.146	0.004	−0.322	− 0.390*	− 0.008
RT	0.173	− 0.018	0.126	0.353	0.153	−0.065	−0.125	0.014
MCI								
DT	0.203	−0.021	−0.209	0.034	0.188	−0.196	−0.103	0.083
RT	−0.002	0.099	−0.039	0.188	0.243	−0.150	−0.084	0.087
NDC								
DT	−0.141	0.332	−0.463	0.139	−0.204	0.201	0.194	0.096
RT	0.119	0.449	−0.128	− 0.281	−0.412	0.090	−0.085	0.133

In all patients, correlations between DT and age and total protein were analyzed using Pearson’s correlation coefficient, and other correlation analyses were conducted using Spearman’s correlation coefficient. In patients with ADD, correlations between DT or RT, and age, BMI, total protein, zinc, and TDAS score were analyzed using Pearson’s correlation coefficient, and other correlation analyses were conducted using Spearman’s correlation coefficient. In patients with MCI, correlations between DT or RT and age, total protein, albumin, and zinc were analyzed using Pearson’s correlation coefficient, and other correlation analyses were conducted using Spearman’s correlation coefficient. In NDCs, correlations between DT and age, medications, salivation, total protein, albumin, and zinc were analyzed using Pearson’s correlation coefficient, and other correlation analyses were conducted using Spearman’s correlation coefficient

*ADD* Alzheimer’s disease dementia, *MCI* mild cognitive impairment, *NDC* non-demented control, *DT* detection threshold, *RT* recognition threshold, *BMI* body mass index, *TDAS* Touch Panel-type Dementia Assessment Scale

Data are presented correlation coefficient (*r*). *p < 0.05

## Discussion

In this study, we comprehensively evaluated taste function in patients with ADD or MCI and in NDCs and showed that the taste recognition thresholds were higher in patients with ADD than in the NDCs. This finding is consistent with the results of other studies [8–10]. In terms of the detection threshold, a study investigating the sweet and sour tastes using a whole-mouth gustatory test [19] reported absence of gustatory alterations in patients with ADD. In contrast, the detection threshold was shown to be increased in patients with ADD in a study on the four basic tastes surveyed by the filter-paper disc method [8, 36]. In this study, we used five basic tastes in 13 different concentrations and found that the overall detection thresholds for various taste solutions showed an increasing trend when we adjusted by age in ADD patients. Therefore, taste detection in the whole oral cavity was considered slightly reduced in ADD patients. In addition, we found no significant differences between the three groups in the comparison of the degree of deviation between the detection and the recognition thresholds, so that the concentration required from detection to recognition did not change, and it seemed that the participants responded to tastes appropriately even in the presence of cognitive function decline. On the other hand, we found no significant taste function disorders in patients with MCI, but another study reported a decrease in taste function in these patients [9]. The authors evaluated each region in the oral cavity, but we evaluated the taste perception in the whole oral cavity, so it is possible that taste perceived in the whole oral cavity (as during a meal) is not decreased with MCI.

In our study, many participants could not recognize umami. The recognition threshold of umami was significantly increased in patients with ADD compared with NDCs. Moreover, 69.0 and 44.2% of patients with ADD and MCI, respectively, could not recognize even the highest concentration of umami solution. This finding suggested the possibility that recognition of umami was the worst in patients with ADD among the three groups. It also suggested that some patients with MCI may have poor recognition of umami. The inability to sense umami is thought to lead to loss of appetite [21], which furthermore leads to a decrease in interest in diet. The amount of overall salivary flow increases with umami taste stimulation by MSG [22], and improving saliva secretion may improve taste perception. In addition, continuous ingestion of MSG has been reported to improve the quality of life and nutritional status of hospitalized elderly patients [37], and to provide benefits to the cognitive function of residents in facilities [32]. Therefore, intervention with MSG for elderly people and patients with dementia is gradually attracting attention. Alternatively, in this study, 21.4% of the participants in the NDC group were not able to recognize even the highest concentration of umami. A previous study reported that at various concentrations of umami, the correct answer rate regarding its perception in the oral cavity was different between young adults and older individuals [38]. However, another report showed that the results of the evaluation of the umami recognition threshold on each region of the tongue did not differ between young adults and older individuals [39]. There is a disagreement regarding the decline in umami recognition with age; nevertheless, the results of our study suggest that umami recognition worsens with age. However, the detection threshold for umami has also been shown to be increased in individuals with dementias other than ADD [40]. Future studies are needed to determine whether this is a specific change in AD, considering its association with pathological changes. For tastes other than umami, the non-recognition percentages for sweet and bitter (even at the maximum concentration) were high in ADD. Overall, more participants did not recognize the taste via G-protein coupled receptors than via ion channels. However, whether this difference in transmission format is associated with a failure caused by disease is unknown and needs future study.

In the dietary and gustatory questionnaires, we found few complaints about the decline in the subjective gustatory function in any group, and many participants answered that they were able to feel the taste of their meals well. Large-scale questionnaires have reported fewer complaints of age-related gustatory dysfunction than of age-related visual, auditory, and olfactory dysfunctions [41], and complete ageusia is rare in subjects with age-related taste function declines [42, 43]. This may be explained by the fact that taste function is unlikely to be impaired because three different cranial nerves (the facial, glossopharyngeal, and vagus nerves) provide sensory coverage for the tongue and mouth. Furthermore, because the decline in taste function due to dementia seems to be slowly progressive, gradually decreasing function may go unnoticed by individuals. In addition, although the clinical examination tested each taste separately, an actual meal consists of several simultaneous tastes. Therefore, discrepancies between the examination and the subjective gustatory function may exist. No significant differences in the results of the dietary and gustatory questionnaire were considered to exist among the three groups for the above reasons. Furthermore, the absence of any dietary problems in the questionnaire suggests that the ADD group, which experienced decreased taste function, did not experience a decrease in BMI due to weight loss. This study was a cross-sectional study, so it is necessary to conduct a longitudinal study. However, even in the absence of subjective symptoms, the test scores may be low in patients with MCI or ADD; thus, accurate assessments are important.

We found a correlation between taste detection and BMI, suggesting that slender individuals may have difficulty perceiving tastes in this study. An association between taste disorders and malnutrition has been reported in the elderly [44], and nutritional bias is thought to lead to zinc deficiency [45] and eventually affect taste. However, there was a large difference in the correlation coefficient between taste detection and BMI among the three groups; MCI and NDCs, had particularly large correlation coefficients. Another factor may be involved in patients with ADD. It is necessary to increase the number of cases to make further conclusions. On the other hand, taste recognition is thought to be associated with age and cognitive function, and cognitive function was not associated with the detection threshold but rather with the recognition threshold. Similar to a previous study [8], the cause of the decline in taste function in dementia is not thought to be due to impaired transmission from peripheral receptors but to a decrease in the taste perception cognitive ability that accompanies brain atrophy and neurodegeneration. However, patients with ADD showed a significant increase in the detection threshold compared with NDCs, so we think that taste detection may be affected by brain disorders. In fact, the insular cortex/frontal operculum has been shown to be activated by water stimulation [46], and some areas of the brain may be involved in taste detection. In addition, a study examining the taste function in patients with semantic dementia, which damages the insular cortex, showed that there is an increase in taste detection thresholds [36]. Moreover, the density of insular neurofibrillary tangles has been significantly positively correlated with the duration of clinical dementia [47].

Many of the participants in this study had decreased salivary secretion as well as low serum zinc levels. The average number of prescription medications in geriatric outpatients at five university hospitals has been estimated to be between 3 and 5 [48]. In this study, the results were roughly similar, and the median of the number of prescription medications was 4–5 in each group of participants. Many individuals were on multiple medications. The decrease in taste function in the elderly is thought to be the result of many factors, including decreased salivary secretion, decreased serum zinc levels, effects of comorbidities, and multidrug use [22, 24, 49]. In this study, we found no significant differences in terms of those variables among the three groups. Therefore, we think the decline in taste function due to AD was caused by factors inherent to the disease and not to age.

Finally, we are aware of the limitations of our study. The first is related to our scoring. Because many participants did not recognize even the maximum concentration of umami, the score reached its peak prematurely, and we cannot rule out that a higher peak may have shown differences between the groups. However, the clinical significance of using a much higher concentration of taste solutions must be carefully considered. The second limitation of our study concerns the age of the participants. Compared with NDCs and patients with MCI, patients with ADD were significantly older. The detection threshold did not significantly differ when we adjusted by age. Gustatory function has been shown to decline with age [38, 42, 50–52], but progression of gustatory hypofunction in elderly (especially after 75 years of age) still remains unclear. We found a correlation between age and taste recognition threshold, but age was also correlated with the cognitive function, and older patients had more severe cognitive disorders. Because older patients have cognitive disorders, it was thought that the gustatory function decreased because of brain disorders that caused cognitive impairment. In addition, there was no significant correlation between age and gustatory test scores in each group, which suggests that aging may be an intermediate factor that promotes factors affecting taste function, such as brain disorders, decreased salivary secretion, decreased serum zinc levels, effects of comorbidities, and multidrug use. All results were to be analyzed by ANCOVA with age as a covariate, but ANCOVA was rarely able to be performed because many results were not normally distributed. The third limitation is that worse gustatory test scores might also be a consequence of cognitive disorders rather than gustatory dysfunction. The presence of multimodal semantic impairment in ADD is well known. Semantic deficits have been reported to potentially reduce olfactory test scores in patients with ADD [3]. In addition, the taste-to-picture matching task score for patients with semantic dementia has been shown to be significantly lower than that of patients with ADD or control patients [36]. Therefore, it is possible that semantic impairment reduced the gustatory test score. However, it was reported that a taste test, in which choices by letter were selected (similar to the method used in this study), showed no significant difference in the scores between patients with semantic dementia and patients with ADD [36], and it is possible that the impact of semantic impairment may be smaller in this study. We believe that, in the future, it will be necessary to verify whether deteriorations in the gustatory test score are due to brain degeneration or cognitive decline. Finally, the number of participants was relatively low. The NDCs were targeted outpatients in the hospital diagnosed as not having dementia, so recruitment was difficult. However, we selected participants according to the exact doctor’s diagnostic criteria, and we believe that the reliability of the results is high.

## Conclusions

In this study, the gustatory function of patients with ADD and MCI, and that of NDCs for five basic tastes was evaluated by the whole-mouth gustatory test. We confirmed taste function declines in patients with ADD, but not in MCI, suggesting the dysfunction of taste is not seen in the early stage of the disease. Salivary secretion, serum zinc levels, and multidrug intake were not correlated with gustatory decline, suggesting that these factors are unlikely to explain our results. However, many patients with ADD or MCI were not able to recognize umami, which decreased their interest in meals, and this variable should be longitudinally studied. Taste function is an indispensable sensory function for adjustment of intake of nutrients necessary for the body and to successfully reject foreign substances. We believe that detecting and interfering with impaired gustatory function at an early stage of recognition impairment may improve dietary interest and quality of life.

## Data Availability

The datasets used and/or analysed during the current study are available from the corresponding author on reasonable request. However, the available of datasets is only possible if all authors and the ethics committee approve.

## Abbreviations

ADD: Alzheimer’s disease dementia
MCI: Mild cognitive impairment
NDCs: Non-demented controls
ADAS-Cog: Alzheimer’s Disease Assessment Scale-Cognitive Subscale
CT: Computed tomography
TDAS: Touch Panel-type Dementia Assessment Scale
BMI: Body mass index
ANOVA: Analysis of variance
ANCOVA: Analysis of covariance
